# CLL kinetics in the tumor microenvironment

**DOI:** 10.18632/oncotarget.21467

**Published:** 2017-10-03

**Authors:** Clare Sun, Adrian Wiestner

**Affiliations:** Clare Sun: Hematology Branch, National Heart, Lung, and Blood Institute, National Institutes of Health, Bethesda, MD, USA

**Keywords:** chronic lymphocytic leukemia, tumor microenvironment, deuterium, cellular proliferation, lymph node

Cumulative evidence points to the importance of the tumor microenvironment (TME) in chronic lymphocytic leukemia (CLL) pathogenesis [1]. *In vitro*, co-culture systems that mimic microenvironmental stimuli are necessary to sustain tumor cells. *In vivo*, engraftment of CLL cells in immunocompromised mice depends on the presence of autologous CD4^+^ T cells. In patients, the effects of the TME on tumor biology have been characterized by gene expression profiling [2]. Peripheral blood (PB), lymph node (LN), and bone marrow (BM) samples were simultaneously obtained from patients with CLL and subjected to whole genome microarray. LN-resident tumor cells showed activation of B-cell receptor (BCR) and nuclear factor (NF)-κB signaling and increased proliferation. The overexpression of proliferation genes in the LN also predicted a shorter time to treatment.

Deuterium (^2^H) is a nonradioactive isotope that can be safely administered in the form of deuterated water (^2^H_2_O) and incorporated into the deoxyribose (dR) moiety of replicating DNA [3]. Isotopic enrichment of dR is measured by gas chromatography mass spectrometric analysis of genomic DNA from the cells of interest. The appearance and loss of newly divided cells over time, or the cell birth and death rates, are calculated from the kinetics of ^2^H enrichment. Deuterated water labeling was first applied in CLL to study the rates of cell birth in PB [4]. The birth rate of tumor cells varied by more than 10-fold between patients, ranging from 0.11% to 1.75% per day. The demonstration of considerable cell proliferation was counterevidence to the historical perspective of CLL as a disease of defective apoptosis leading to cell accumulation.

To investigate tumor cell kinetics in the TME, we used deuterium labeling to measure cell birth and death rates in the PB, LN, and BM of patients with CLL [5]. The birth rate and fraction of newly divided cells were highest in the LN compared to PB or BM. These findings were corroborated with flow cytometric analysis of Ki67 expression, demonstrating the LN as the primary site of tumor proliferation. Moreover, the birth rate in LN, but not PB or BM, was inversely correlated with lymphocyte doubling time and time to first treatment.

It was recently shown that dim expression of the C-X-C motif receptor 4 (CXCR4) together with bright expression of CD5 on flow cytometry identifies a fraction of CLL cells in the PB that are activated and thought to have recently emigrated from the LN. Using deuterium labeling, we found the highest proportion of newly divided cells within the CXCR4^dim^CD5^bright^ population not only in the PB, but also in the LN. Therefore, we conclude that this immunophenotype identifies a distinct subpopulation with the greatest proliferative activity irrespective of location.

Deuterium labeling has also been used to study lymphocyte kinetics during treatment with ibrutinib. Ibrutinib therapy decreased the cell birth rate and accelerated the disappearance of cells from the PB of patients with CLL [6]. These findings were consistent with earlier data that showed an immediate decrease in proliferation and an increase in apoptosis of CLL cells on ibrutinib [7]. However, because deuterium incorporation was only measured in circulating cells, further studies are required to determine how ibrutinib affects the rate of cell turnover in different anatomic sites. Deuterium labeling represents a powerful tool that could be used to address this question and to evaluate tumor kinetics with other novel therapies.

## References

[1] Herman SE (2016). Semin Oncol.

[2] Herishanu Y (2011). Blood.

[3] Busch R (2007). Nat Protoc.

[4] Messmer BT (2005). J Clin Invest.

[5] Herndon TM (2017). Leukemia.

[6] Burger JA (2017). JCI Insight.

[7] Herman SE (2014). Leukemia.

